# Inappropriate cathepsin K secretion promotes its enzymatic activation driving heart and valve malformation

**DOI:** 10.1172/jci.insight.133019

**Published:** 2020-10-15

**Authors:** Po-Nien Lu, Trevor Moreland, Courtney J. Christian, Troy C. Lund, Richard A. Steet, Heather Flanagan-Steet

**Affiliations:** 1 Greenwood Genetic Center, J.C. Self Research Institute, Greenwood, South Carolina, USA.; 2 Biochemistry, Cell and Developmental Biology, Emory University Laney Graduate School, Atlanta, Georgia, USA.; 3 Department of Pediatrics, University of Minnesota Medical School, Minneapolis, Minnesota, USA.

**Keywords:** Cell Biology, Development, Cardiovascular disease, Lysosomes, Proteases

## Abstract

Although congenital heart defects (CHDs) represent the most common birth defect, a comprehensive understanding of disease etiology remains unknown. This is further complicated since CHDs can occur in isolation or as a feature of another disorder. Analyzing disorders with associated CHDs provides a powerful platform to identify primary pathogenic mechanisms driving disease. Aberrant localization and expression of cathepsin proteases can perpetuate later-stage heart diseases, but their contribution toward CHDs is unclear. To investigate the contribution of cathepsins during cardiovascular development and congenital disease, we analyzed the pathogenesis of cardiac defects in zebrafish models of the lysosomal storage disorder mucolipidosis II (MLII). MLII is caused by mutations in the GlcNAc-1-phosphotransferase enzyme (Gnptab) that disrupt carbohydrate-dependent sorting of lysosomal enzymes. Without Gnptab, lysosomal hydrolases, including cathepsin proteases, are inappropriately secreted. Analyses of heart development in *gnptab*-deficient zebrafish show cathepsin K secretion increases its activity, disrupts TGF-β–related signaling, and alters myocardial and valvular formation. Importantly, cathepsin K inhibition restored normal heart and valve development in MLII embryos. Collectively, these data identify mislocalized cathepsin K as an initiator of cardiac disease in this lysosomal disorder and establish cathepsin inhibition as a viable therapeutic strategy.

## Abstract 2

Mislocalized cathepsin K promotes cardiac disease in a zebrafish model of the lysosomal disorder mucolipidosis II and can be targeted by cathespin inhibition.

## Graphical abstract

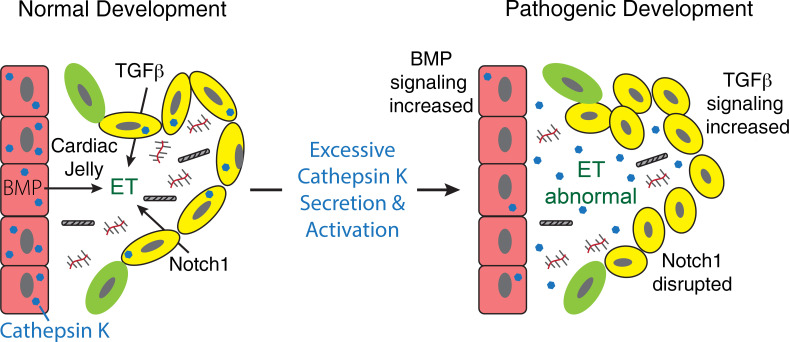

## Introduction

Congenital heart defects (CHDs) represent the most common birth defect, affecting ~40,000 live births annually in the US (1, 2). While the etiology of many of these defects is unknown, studies of inherited genetic disorders with associated CHDs have provided important insight into the mechanisms that drive cardiovascular development and disease (3–5). The lysosomal storage disorders (LSDs), genetic diseases that impair either lysosome biogenesis or the catabolic destruction of lysosomal substrates, frequently exhibit cardiac defects (6–10). One such disorder, mucolipidosis II (MLII), is commonly associated with cardiomyopathy, mitral valve prolapse, and aortic regurgitation (11–13). Furthermore, many children with the attenuated disorder, MLIII, have undergone valve replacement surgery (14, 15). MLII is caused by loss of mannose-6-phosphate–dependent (M6P-dependent) lysosomal targeting (16, 17). In MLII mutations in the GNPTAB gene, encoding the catalytic subunits of the GlcNAc-1-phosphotransferase (GNPT) enzyme, impair addition of M6P residues onto N-glycans of lysosomal hydrolases (18, 19). Without M6P, most soluble hydrolases, including the cathepsin proteases, are hypersecreted from cells.

Cathepsin proteases are lysosomal enzymes whose diverse functions range from protein turnover and hormone or neuropeptide processing to modulation of growth factor activation and stability (20–23). Several cathepsin proteases, namely B, K, L, and S, have been implicated in onset of a variety of cardiovascular diseases (24–26). Increased expression of cathepsins K, L, and S is associated with atherosclerosis, cardiac hypertrophy, and valvular stenosis (7, 27–29), with several studies suggesting cathepsin deficiency protects against these forms of cardiac dysfunction (30, 31). A role for cathepsins K and L has also been shown during heart regeneration and valvular remodeling (24). Although cathepsin proteases are known to be essential for tissue remodeling and can clearly perpetuate later-stage disease, their role during cardiovascular development remains uncertain. Mouse models of cathepsin deficiency have not revealed any obvious defects in the formation of myocardial or valvular tissue, but overlap in the expression patterns and substrate specificities of different cysteine cathepsins could mean compensation occurs in the mouse.

Since aberrant cathepsin expression is associated with later-stage cardiovascular diseases and secretion of cathepsin proteases has been linked with their increased activity, here we asked whether loss of *gnptab*, which controls lysosomal targeting of cathepsin proteases, impairs early cardiovascular development (20, 21, 32). This is important clinically, since children with mutations in GNPTAB often die from cardiac complications, and it is not known whether cardiac features associated with MLII stem from undiagnosed developmental defects. Using TALEN, we generated stable zebrafish lines carrying null mutations in *gnptab*. *gnptab*^–/–^ embryos exhibit pronounced cardiac edema resulting from deficits in both myocardial development and atrioventricular (AV) valve formation. Molecular and phenotypic analyses of cardiac development in the *gnptab*^–/–^ embryos revealed abnormally widespread cathepsin activity throughout MLII hearts and valves. Altered cathepsin activity was associated with reduced BMP and increased TGF-β signaling, both of which are essential for early heart formation. Remarkably, we show that treatment with the cathepsin K–specific inhibitor odanacatib rescues defects in growth factor signaling and restores normal morphology and function to MLII hearts. Moreover, altered heart and valve development was also noted in a transgenic line expressing a secreted variant of cathepsin K. Collectively, these data identify mislocalized cathepsin K as an initiator of the cardiac disease associated with this lysosomal disorder and establish cathepsin inhibition as a viable therapeutic strategy.

## Results

### TALEN-mediated loss of the GNPT enzyme expression causes cardiac anomalies.

**Figure 1:**
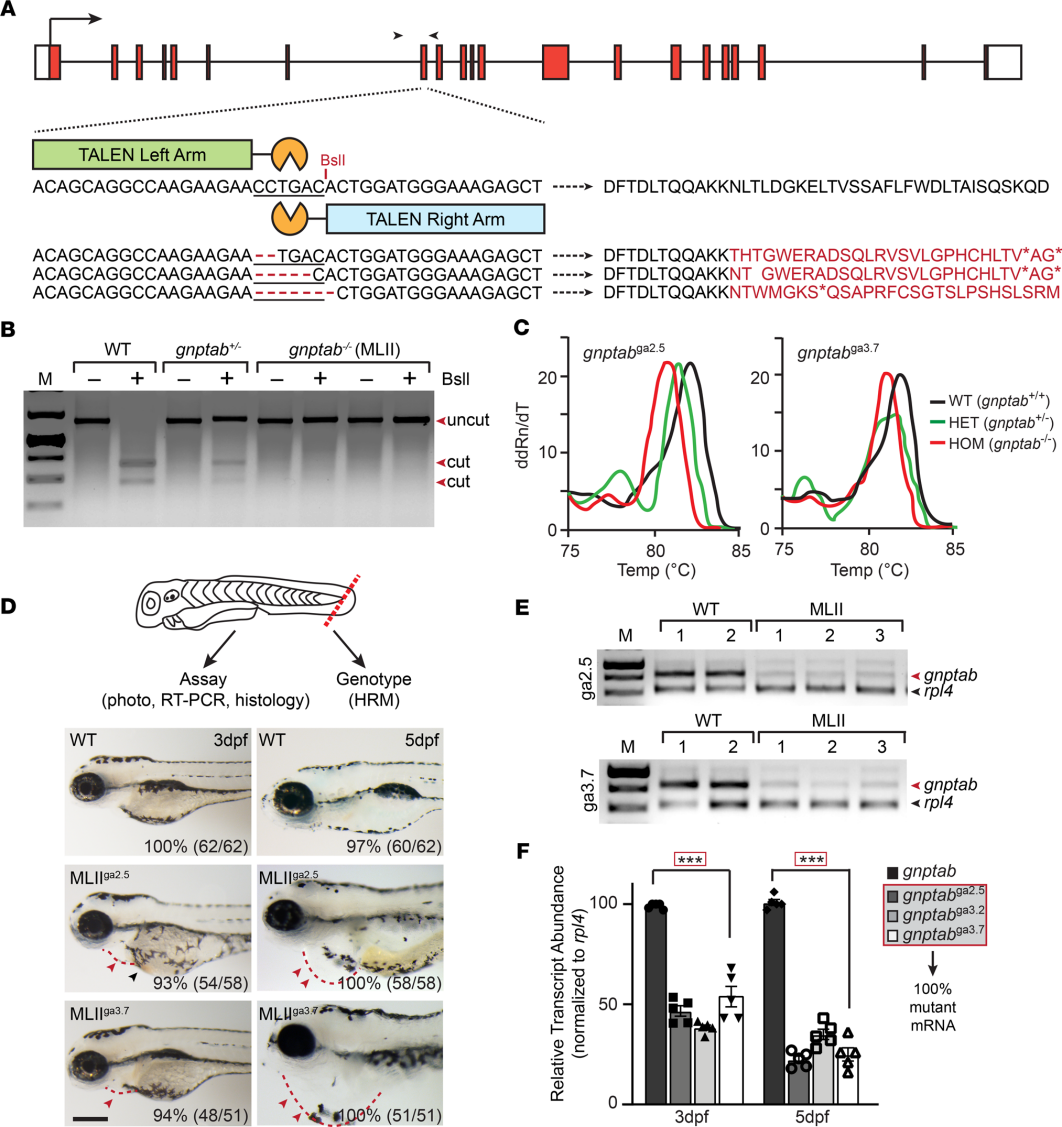
TALEN-mediated KO of *gnptab* disrupts cardiovascular development. (**A**) Schematic of *gnptab* gene shows the locations of the left and right TALEN arms, the PCR primers used for genotyping (black arrowheads), and 3 isolated zebrafish lines carrying 2, 5, and 7 bp frame-shifting deletions. The predicted “translation” of these products is listed. (**B**) BslI digestion of genomic DNA identifies *gnptab* WT (+/+), heterozygous (+/–), and homozygous mutant (–/–, MLII) animals. (**C**) High-resolution melt analyses yields unique patterns that confirm the 3 expected genotypes. (**D**) Schematic illustrates live embryo dissections used in HRM analyses to assign the genotypes before experiments. Images of 3- and 5-dpf-old WT and MLII (*gnptab^–/–^*) animals from lines carrying 5 and 7 bp deletions (*gnptab*^ga2.5^ and *gnptab*^ga3.7^) show progressive cardiac edema. Percent values equal the number of embryos exhibiting phenotypes similar to the picture. *n* = 50–60 embryos from 4–5 independent matings per line. Scale bar: 100 μm. Red arrowheads indicate edema; black arrowhead indicates pooled blood. (**E**) RT-PCR analyses of *gnptab* expression of embryos from 2 pools of WT and 3 pools of 5 bp–deleted and 7 bp–deleted embryos show reduced transcript abundance. Analyses of *rpl4* transcripts provides an internal reference. Representative gel of 4 independent experiments. (**F**) Quantitation of transcript abundance show 60%–75% reduction in MLII lines from 3 to 5 dpf. Gel extraction and sequencing show 100% of residual transcripts in the mutant lines are mutant mRNAs. *n* = 100 embryos from 4 experiments, with 20 cloned transcripts sequenced per condition. ***P < 0.001, Dunnett’s test with correction.

MLII GNPTAB^–/–^ patients commonly exhibit systolic murmurs, cardiomegaly, mitral valve prolapse, and aortic regurgitation (11–13). A role for the GNPT in cardiac development is further supported by the fact that morpholino inhibition of its expression causes a prominent cardiac edema in zebrafish embryos (33). To investigate the pathogenic mechanisms driving MLII cardiac pathology, *gnptab*-null zebrafish were generated using TALE nucleases that target exon 8, directly upstream of the catalytic domain (Figure 1A and Supplemental Figure 1; supplemental material available online with this article; https://doi.org/10.1172/jci.insight.133019DS1). TALEN-injected adults carrying 2–7 bp frame shifting deletions were isolated and outcrossed over 4 generations with age-matched TLAB animals. Founders carrying mutations in *gnptab* were initially identified using BslI enzyme digestion of genomic PCR products generated from F1 outcross progeny (Figure1B). DNA fragments not cleaved by BslI were cloned and sequenced. Stable lines were generated from animals carrying 2, 5, and 7 bp deletions in the *gnptab* coding sequence (Figure 1A). Both BslI digestion patterns and high-resolution melting (HRM) curves were used to assign adult and embryonic genotypes in all subsequent generations (Figure 1, B and C). Prior to all molecular and biochemical analyses, the progeny of *gnptab*^+/–^ matings were genotyped using a microdissected piece of the tail fin (Figure 1D). Embryos were then either individually studied or pooled by genotype and collectively analyzed.

Crosses of F4 generation *gnptab*^+/–^ adults yielded progeny of all 3 genotypes in expected Mendelian ratios (Supplemental Figure 1). Like previously noted in *gnptab* morphants, the *gnptab*^–/–^ progeny exhibited cardiac edema, round neurocraniums, retracted jaws, and a pronounced motility defect (Figure 1D). The cardiac phenotype and associated edema typically appeared ~3 days postfertilization (dpf), becoming progressively more severe 5–6 dpf. As a result, the majority of affected embryos died 5–7 dpf (Supplemental Figure 1). A combination of conventional reverse transcription PCR (RT-PCR) and quantitative PCR (qPCR) confirm that the 2, 5, and 7 bp mutations generated null alleles in these TALEN-injected lines (Figure 1, E and F). *gnptab*^–/–^ embryos not only express significantly less phosphotransferase mRNA than control embryos, but sequencing of cloned cDNAs demonstrate that 100% of the mRNA expressed in 3 and 5 dpf mutant embryos is derived from the mutant alleles. Because all 3 deletions introduce multiple in-frame stop codons, *gnptab*^–/–^ is considered phosphotransferase null. Although it is unclear why the mRNA transcripts containing frame-shifting deletions are less abundant than WT transcripts, their loss may occur either by nonsense-mediated decay or suppressed expression of the mutant allele. Both mechanisms have been documented as quality control processes that protect cells from translating and accumulating aberrant transcripts (34–36). Alternatively, the apparent reduction in abundance of mutant transcripts could also reflect sequence-specific differences in PCR amplification.

### Myocardial and AV valve formation are disrupted in gnptab^–/–^ embryos.

**Figure 2:**
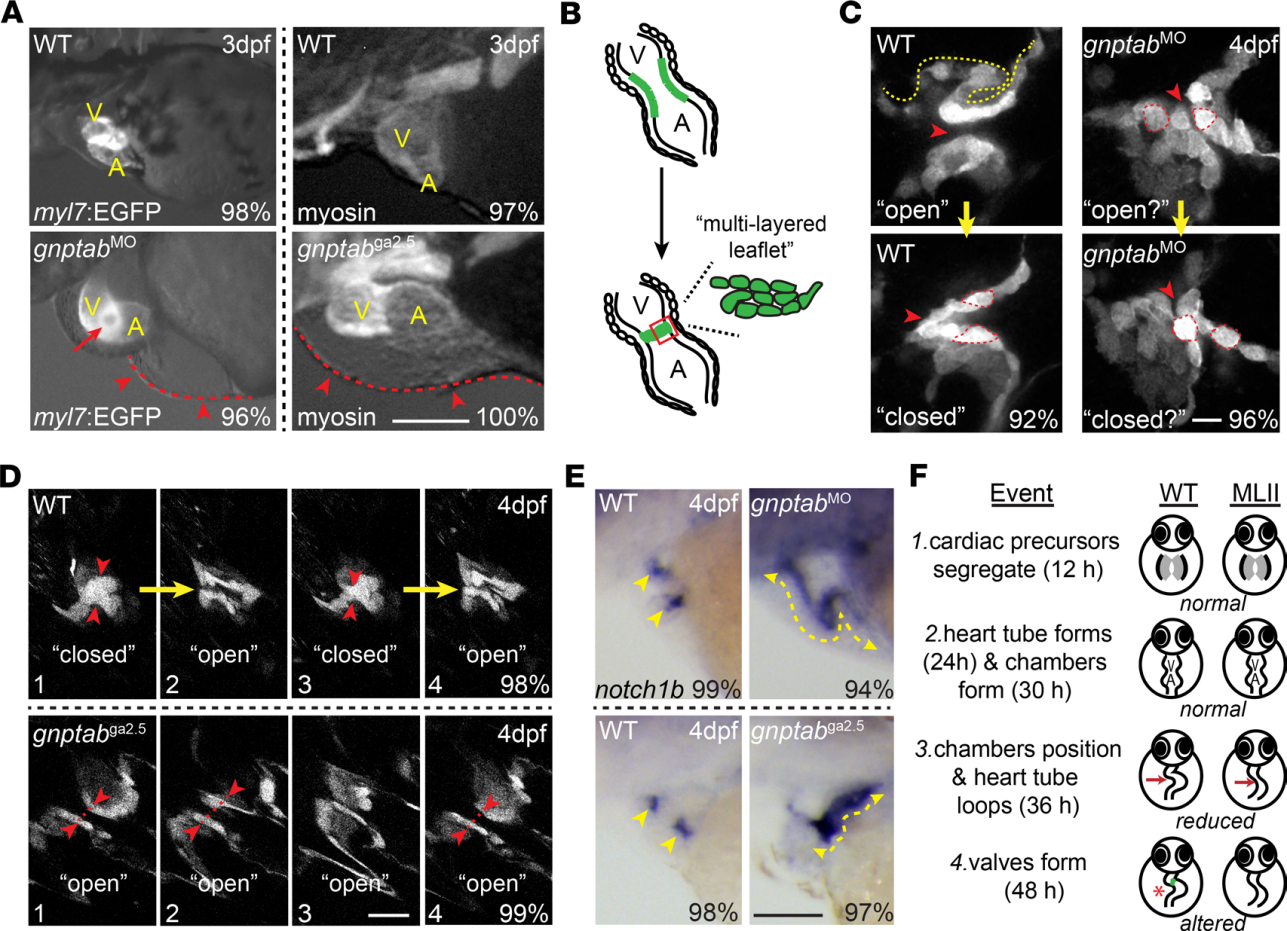
Loss of *gnptab* gene expression disrupts heart looping and AV valve formation. (**A**) Fluorescent stereoscopic images of 3 dpf WT and *gnptab* morphants (MO) in the *myl7*:EGFP (labels cardiomyocytes) background show reduced looping and inferior edema (red arrow) in MLII hearts. Red arrowheads highlight edema. Similar phenotypes are noted in *gnptab* mutants (ga2.5 shown) stained immunohistochemically for myosin. Scale bar: 50μm. V, ventricle; A, atrium. Percent values equal the number of embryos exhibiting phenotypes similar to the picture. *n* = 30 embryos from 4–5 independent matings. (**B**) Schematic of AV valve formation. (**C**) Live confocal imaging of *tie2*:EGFP^+^ hearts reveal abnormal uncondensed valves in *gnptab* morphants that do not open and close as the heart beats. *n* = 35–40 total embryos from 3 independent matings. Scale bar: 10 μm. Red arrowheads highlight the canal between the left and right sides of the valve. (**D**) Live confocal imaging of *tie2*:EGFP^+^ *gnptab* mutant (ga2.5 shown) valves show similar disruption in architecture and behavior. Images 1–4 show WT valves opening and closing at regular intervals, while the *gnptab*/MLII mutant valves remain open in images 1,2, and 4. Scale bar: 20μm. *n* = 25 embryos from 3 matings. Red arrowheads highlight left and right sides of the valve, which fully “close” in WT but not mutant embryos. (**E**) In situ analyses of *notch1b* transcripts show that differentiation of endocardial and AV valve cells is disrupted in both *gnptab* morphants and mutants, with expression present throughout the endocardium (yellow lines) instead of restricted to valvular regions (yellow arrow heads). Scale bar: 50μm. Percent values equal the number of animals with pictured phenotype. *n* = 75 embryos from 3 experiments. (**F**) Schematic illustrates key aspects of early heart and valve development and summarizes the events disrupted in MLII embryos.

Gross microscopic analyses of EGFP-labeled and myosin-stained hearts demonstrate that *gnptab*^–/–^ mutant and morphant embryos exhibit similar cardiac anomalies (Figure 2A). These include pericardial edema, alterations in the size and shape of the atrial and ventricular chambers, and disrupted heart tube morphogenesis. Although mutant and morphant embryos assemble a linear tube with identifiable chambers, both hearts lack the full rotation that normally positions the atrium behind the ventricle. Both also exhibit a loss of unidirectional blood flow, where regurgitation between the chambers causes blood to pool at the heart’s base (see red arrowheads, Figure 2A). Stereoscopic images of EGFP-labeled (*myl7*:EGFP) hearts also revealed an opening at the AV boundary, which was present in 96% of *gnptab*^–/–^ embryos imaged (see red arrow, Figure 2A). This finding suggested that altered blood flow may stem from malformed AV valves.

Normally, between 48 and 60 hours postfertilization (hpf), a subset of endocardial cells transition from a single cuboidal layer into a compact cellular structure clustered at the AV boundary (Figure 2B; ref. 37). In fish, this structure forms when the AV endocardial cells elongate, invade the extracellular matrix, and collectively migrate to form multilayered leaflets (38, 39). Live confocal analyses using the *tie2*:EGFP transgenic line (40), which labels endocardial valvular cells, show alterations in the shape, organization, and behavior of *gnptab*-deficient AV cells. Unlike WT AV cells, *gnptab*-deficient cells remain cube-shaped and fail to form a discretely layered structure (see yellow line denoting layers and outlined cells denoting shape, Figure 2C). Furthermore, while WT valves open and close as the heart contracts, *gnptab*-deficient valvular tissues do not dynamically move when the heart beats (Figure 2C). Failure to open and close synchronously with contraction allows the blood to flow backward. Similar disruptions in AV cell behavior were also noted in *tie2*:EGFP;*gnptab*^ga2.5^ mutant embryos (Figure 2D). To ask whether the disrupted valvular morphology noted in *gnptab*^ga2.5^ mutant and morphant embryos reflects a defect in the differentiation of valve precursor cells (VPCs), we performed in situ analyses for the endocardial marker *notch1b* (Figure 2E). In WT, *notch1b* expression is initially noted throughout the entire endocardium but progressively restricts to the outflow tract and AV valves. Localizing Notch signaling to the VPCs is essential for their transition from endocardium to specified valvular cells (41). In both the *gnptab* mutant and morphant embryos, *notch1b* expression remains high in nonvalve endocardial cells, indicating a disruption in VPC maturation. Collectively, these data suggest that, although the earliest aspects of heart development are largely normal in animals lacking GNPT, later-stage events including proper heart tube looping and valve formation are disrupted (Figure 2F). Since cardiac morphogenesis is affected, it is currently unclear whether the valvular anomalies noted in *gnptab*-deficient (MLII) embryos occur cell autonomously or whether reduced myocardial rotation, associated reductions in blood pressure, or altered contractile force also contribute to onset of the valvular phenotype.

### TGF-β and BMP signals are disrupted in gnptab^–/–^ hearts.

**Figure 3:**
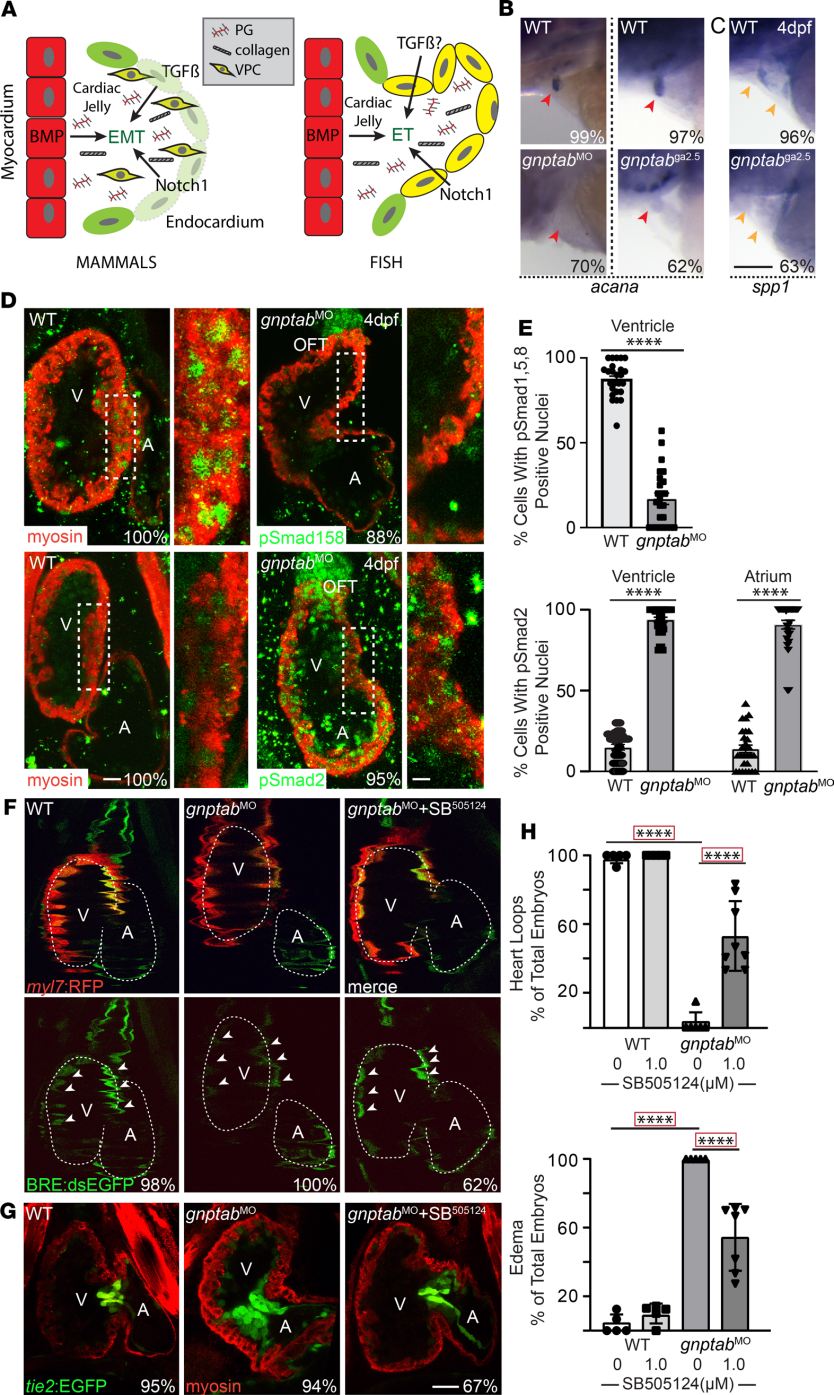
Disruptions in BMP and TGF-β signaling are associated with MLII heart and valve defects. (**A**) Schematic illustrates role for TGF-β, BMP, and Notch signals during AV valve development comparing mammals and fish. PG, proteoglycan; VPC, valve precursor cell; EMT, endocardial to mesenchymal transition. (**B** and **C**) In situ analyses of aggrecan (*acana,* red arrowheads) (**B**) and osteopontin (*spp1,* yellow arrowheads) (**C**) in 4 dpf embryos show defects in expression of the cardiac jelly are associated with loss of mesenchymal migration in *gnptab*/MLII morphants and mutants. Percent values indicate the number of embryos with the pictured phenotype. *n* = 50–65 embryos from 3 experiments. Scale bar: 50 μm. (**D**) Confocal images of WT and *gnptab* morphants stained immunohistochemically for myosin (red) and either pSmad1/5/8 or pSmad2 (green) illustrate reduced BMP and increased TGF-β signaling in MLII hearts. Boxed areas highlight region particularly affected, which are magnified in panels to the right. V, ventricle; A, atrium; OFT, outflow tract. *n* = 25 embryos from 3 independent matings. Scale bar: 20 μm. (**E**) Graphs show the percentage of cells in the ventricle and atrium with nuclear localized pSmad2 and pSmad1/5/8 staining. Data are presented as mean ± SEM. ****P < 0.0001 using 2-tailed Student’s *t* test. (**F**) Live confocal images of 4 dpf embryos expressing the BRE:dsEGFP (reports BMP signaling, denoted by white arrowheads) and *myl7*:RFP (labels cardiomyocytes red) confirm reduced BMP signaling (labeled green by BRE:dsEGPP) in the ventricle of MLII hearts, which is restored when TGF-β signaling is inhibited with SB505124. *n* = 10–12 embryos from 3 independent experiments. (**G**) Confocal images of *tie2*:EGFP^+^ (green) WT, *gnptab*-deficient morphants, and TGF-β–inhibited *gnptab*-deficient morphants treated with SB505124. Embryos stained immunohistochemically for myosin (red). *n* = 25–30 embryos from 3 independent experiments. Scale bar: 30 μm. (**H**) Graphs show percent of embryos whose hearts loop normally and that exhibit edema. For simplicity, each dot represents the average value obtained from an experiment containing 25–30 embryos. Total *n* > 100 embryo per condition. Data represent mean ± SEM. ****P < 0.0001 using Dunnett’s test with correction.

In mammals, proper valve formation depends on the coordinated action of the TGF-β, BMP, and Notch signaling cascades (Figure 3A; ref. 42). Initially, BMP signals emanating from the myocardium induce the endocardium to secrete a specialized matrix termed the cardiac jelly (Figure 3A). This matrix, which is rich in proteoglycans and collagen, functions as a cushion between the 2 layers, where VPCs embed themselves following an endothelial to mesenchymal transition (EMT) (43). In fish, rather than a complete mesenchymal transition, this process initially occurs when cells collectively migrate, causing AV endocardial tissue to involute (Figure 3A, fish) (38, 44). Since confocal analyses indicate that *gnptab*^ga2.5^ mutant VPCs are disorganized and lack identifiable layers, we assessed expression of 1 proteoglycan thought important for VPC condensation, aggrecan (*acana*). In situ analyses of *acana* mRNA showed that, unlike control embryos, which exhibit strong expression in outflow tract valves of 4 dpf embryos, *acana* mRNA is virtually absent from both *gnptab*^–/–^ mutant and morphant valves (Figure 3B). Loss of *acana* expression was matched by a decrease in osteopontin (*spp1*), which is typically expressed by migrating mesenchymal cells, including involuting VPCs. Reduced *spp1* expression was apparent in both the outflow tract and AV valves of *gnptab^–/–^* embryos (Figure 3C). Collectively, these data suggest that mutation of *gnptab* alters cardiac jelly composition, in turn compromising VPC maturation and condensation.

Studies in multiple systems suggest that synergy between the TGF-β and BMP pathways regulates expression of essential matrix components like *acana* (42). TGF-β signaling also plays a central role in many EMT events (45). However, in fish, since VPCs do not undergo true EMT and fully delaminate, it is unclear to what degree TGF-β signals contribute to this aspect of valvular formation (Figure 3A). To ask if either signaling system is altered in the *gnptab*^ga2.5^ mutant hearts, embryos were stained immunohistochemically for the transcriptionally active forms of the BMP and TGF-β effectors phospho-Smad1/5/8 (pSmad1/5/8) and pSmad2, respectively. Confocal analyses demonstrate that the number of myocardial cells containing pSmad1/5/8^+^ nuclei was reduced by 70%, while pSmad2^+^ nuclei was increased nearly 80% in *gnptab* morphant embryos (Figure 3, D and E). Altered activation of pSmad2 was noted throughout the myocardium of both the ventricle and atrium. The degree of altered BMP activity was further evaluated in animals carrying *cmlc(myl7)*:RFP (labels myocardium) and BRE:dsEGFP transgenes (Figure 3F and Supplemental Figure 2). BRE:dsEGFP transgenic animals express a destabilized (ds) form of EGFP in response to BMP growth factor signals. Live confocal analyses of embryos 4 dpf show that, in WT, BMP activity is typically high on the right side of the superior ventricle but lower on the left side. BMP activity in both regions was reduced (relative to myosin expression) ~50% in *gnptab*^–/–^ morphants (Figure 3F and Supplemental Figure 2). Due to significant crosstalk between the TGF-β and BMP signaling systems, high TGF-β activity has been shown to negatively impact BMP activity (46, 47). To address this possibility, *gnptab*^–/–^ morphants were treated at 52 hpf with the TGFBRI inhibitor SB505124 (10 μM). Inhibition of TGF-β signaling recovered normal patterns of BMP activation in 62% of the drug-treated MLII embryos (Figure 3F and Supplemental Figure 2), with BMP-driven EGFP fluorescence increasing ~30% following drug treatment. Confocal analyses of *gnptab* morphant embryos treated with SB505124 showed that inhibiting TGF-β signaling also substantially improved AV valve morphology and chamber rotation (heart looping) and reduced edema in morphant hearts (Figure 3, G and H). These findings suggest that disruptions in TGF-β–related signaling may underlie multiple aspects of MLII cardiac pathology.

### Cathepsin protease activity is altered in gnptab-deficient hearts.

**Figure 4:**
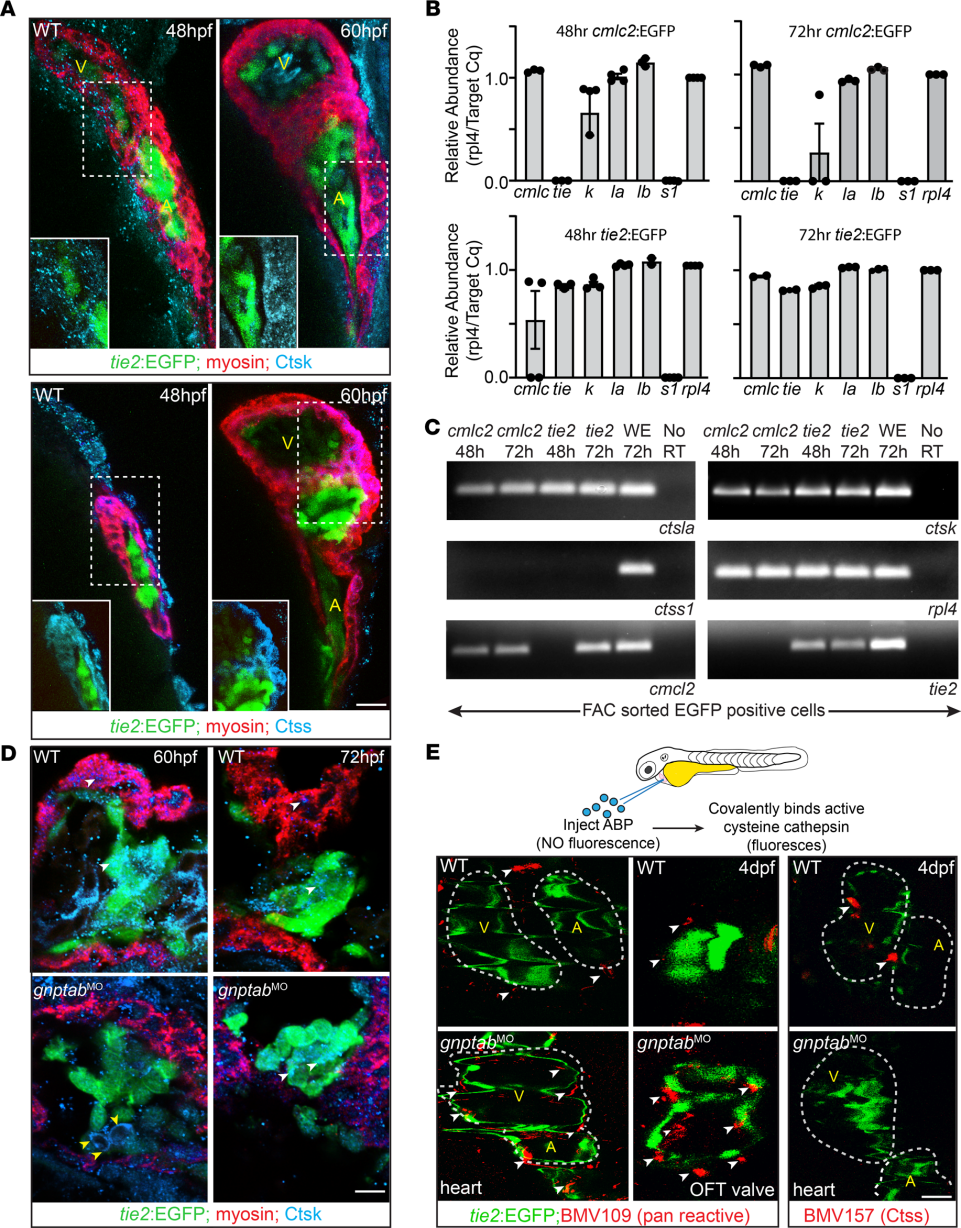
Cathepsin proteases are expressed in developing hearts. (**A**) Confocal images of 48–60 hpf *tie2*:EGFP^+^ (labels valves green) hearts stained immunohistochemically for myosin (red) and either Ctsk or Ctss (blue) show cathepsin proteases are expressed in multiple regions of heart, with Ctsk enriched in cardiomyocytes (see insets of boxed regions) throughout ventricle and atria and Ctss predominantly present in region between the 2 chambers (see inset of boxed regions). *n* = 15–25 embryos imaged per condition. Scale bar: 20μm. V, ventricle; A, atrium. (**B**) qPCR of FAC sorted EGFP^+^ cardiomyocytes (*cmlc*:EGFP) and endocardial (*tie2*:EGFP) cells. Transcript abundance calculated relative to *rpl4*. Data are presented as mean ± SEM (**C**) Gel-based RT-PCR analyses of cathepsin expression in FACS-sorted cells. *n* = 50–100 embryos per sort. Representative gel from 3–4 independent experiments. (**D**) Confocal images of cryosections from 60–72 hpf *tie2*:EGFP^+^ (labels valves green) hearts stained immunohistochemically for myosin (red) and Ctsk (blue). *n* = 15 embryos imaged per condition. Scale bar: 20 μm. (**E**) Schematic illustrates cardiac injection of an activity-based probe (ABP), which does not fluoresce until covalently bound to an activated cysteine cathepsin. Confocal images of WT and *gnptab* morphants (MO) labeled with the pan reactive probe BMV109 reveal generally increased cathepsin activity (red, denoted by white arrowheads) present throughout the MLII heart. Injection of the Ctss-specific probe BMV157 (red) shows its activity is reduced in the MLII heart compared with other cathepsins, likely K and L. Representative images from 3 independent experiments, *n* = 30 embryos per condition. Scale bar: 30 μm. OFT, outflow tract.

Previous studies in *gnptab-*deficient embryos showed that loss of M6P causes cathepsin proteases to be mislocalized outside of cells, increasing their activity and driving pathology in MLII cartilage (20, 21, 32). Increased activity of Ctsk and Ctsl was demonstrated in *gnptab*-deficient embryos using the cathepsin-specific activity-based probed (ABP) BMV109. BMV109 contains a Cy5 moiety whose fluorescence is quenched until the probe covalently binds activated cysteine cathepsins (48). In addition to increases in the activities of Ctsk and Ctsl, global analyses also revealed an MLII-associated decrease in Ctss activity (21). Immunohistochemical staining and transcript analyses indicate that Ctsk, Ctsl, and Ctss are all expressed in the zebrafish heart from 48 to 72 hpf (Figure 4, A–C). Confocal images of embryos stained in whole mount show Ctsk and Ctsl (not shown) expression in both myocardial and valvular cells (Figure 4A). Although Ctss was also detected in the myocardium, it was restricted to the lateral regions closest to the AV junction (Figure 4A). To independently confirm cathepsin expression in these tissues, *cmlc*:EGFP and *tie2*:EGFP transgenic embryos were dissociated into single cells and EGFP^+^ myocardial or valvular cells isolated by FACS. Both qPCR and endpoint RT-PCR performed on RNA from cells isolated at 48 and 72 hpf detected Ctsk and Ctsl transcripts, but not Ctss (Figure 4, B and C). The inability to detect Ctss transcripts may reflect the fact that its expression appears to be restricted to a small number of cells. Importantly, while transcript analyses on cells isolated 72 hpf consistently detected *ctsk* transcript in valvular populations, its expression was variable in cardiomyocytes, with low transcript levels only noted in 50% of the populations analyzed. These data suggest that *ctsk* transcript wanes in cardiomyocytes 72 hpf. To further evaluate cathepsin expression and assess whether enzyme localization is altered in the MLII heart, cryosections of *tie2*:EGFP^+^ embryos were also stained immunohistochemically. Confocal images of embryonic sections stained for Ctsk show similar expression patterns to that noted in whole mount, with Ctsk detected in both myocardial and valvular cells (Figure 4D). As previously shown in cartilage, overall tissue distribution was similar between control and *gnptab*-deficient hearts. Subtle differences were, however, noted in Ctsk’s subcellular localization. In particular, Ctsk was often found evenly distributed around the perimeter of MLII valve cells (Figure 4D, yellow arrowheads). Pericellular distribution, which was also previously observed in *gnptab*-deficient chondrocytes, was not noted in control embryos.

To additionally ask whether the activity of cathepsin proteases is locally altered within the MLII heart, the BMV109 probe was injected into the pericardial space of 3.5 dpf embryos, and protease activity was assessed visually 12 hours after injection (Figure 4E). Live confocal images showed that — unlike WT hearts, where discrete pockets of cathepsin activity were noted in the atria, the ventricle, and the outflow tracts (OFT) — MLII hearts exhibit substantially more activity in each of these regions (white arrowheads, Figure 4E). Protease activity was particularly high in the OFT of the *gnptab*-deficient embryos. Although these images do not indicate whether the MLII-associated increases in cathepsin activity occur intra- or extracellularly, previous studies in dissociated cells indicate the majority of Ctsk and Ctsl’s activity exists outside MLII cells (20). This is consistent with the pericellular localization noted in valvular cells of stained sections (Figure 4D, yellow arrowheads). Because BMV109 reacts with all mature cysteine cathepsins, we also used a second ABP (BMV157) that only binds active forms of Ctss (49). Unlike seen with the pan-specific ABP (BMV109), BMV157 labeling revealed 2 discrete regions of the Ctss activity in WT 4 dpf but very little Ctss activity in either MLII morphants or mutants (Figure 4E). The similarity between BMV157 labeling and immunohistochemical analyses of Ctss suggests it is indeed expressed but at levels below our detection in pools of sorted cells. Collectively, these data suggest that, while *gnptab* deficiency increases the activity of Ctsk and Ctsl, loss of M6P biosynthesis may actually reduce Ctss activity.

### Inhibiting cathepsin K’s activity ameliorates multiple aspects of MLII heart pathology.

**Figure 5:**
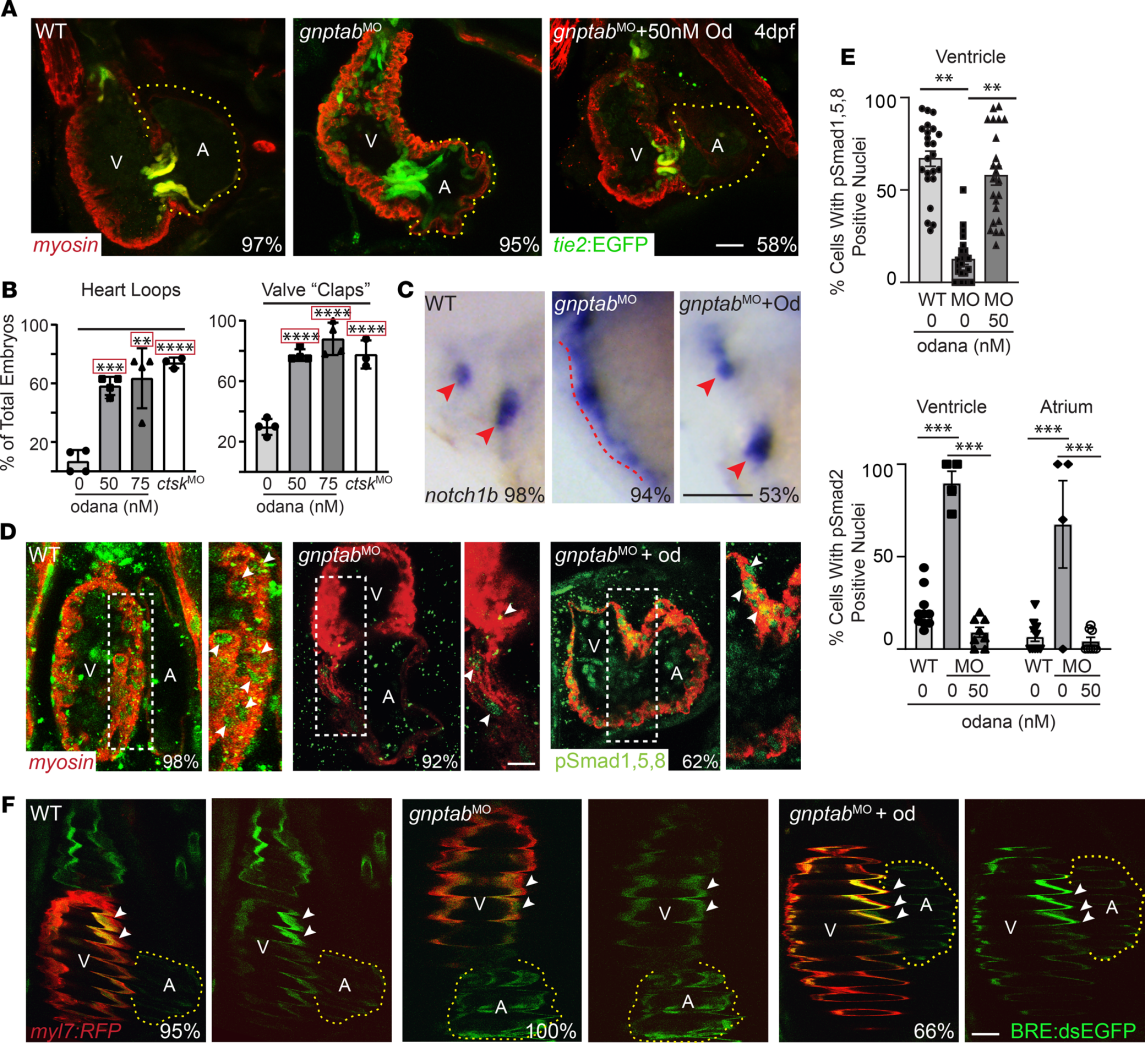
Treatment with the cathepsin K inhibitor odanacatib rescues MLII heart defects. (**A** and **B**) Confocal analyses of *tie2*:EGFP^+^ (green) hearts stained immunohistochemically for myosin (red) show that treatment with 50 nM odanacatib restores normal morphology to MLII hearts and valves, as assayed by the number of MLII embryos with hearts that loop and valves that open and close (“clap”) at regular intervals following treatment. Percent values on images represent the number of embryos with the pictured phenotype. *n* = 100–125 embryos. Data are presented as mean ± SEM. **P < 0.01,***P < 0.001, ****P < 0.0001 using Dunnett’s test with correction (indicated by red box). Each dot represents the average of 20–25 embryos from 4–5 experiments. Scale bar: 25 μm. (**C**) In situ analyses of *notch1b* expression show restored differentiation of MLII valve cells (red arrowheads) following treatment with odanacatib. Percent values represent the number of embryos with the pictured phenotype. *n* = 75 embryos from 3 experiments. Scale bar: 50 μm. (**D**) Confocal analyses of embryos stained immunhistochemically for myosin (red) and pSmad1/5/8 (green) show odanacatib treatment increases BMP signaling in MLII embryos. Panels to the right represent higher-power images of boxed regions; white arrowheads denote nuclear localized pSmad1/5/8. Scale bar: 20 μm. (**E**) Graphs show percent cells containing Smad^+^ nuclei in WT, *gnptab* morphant, and odanacatib-treated embryos. *n* = 20–25 embryos imaged from 3 experiments. Data are presented as mean ± SEM. Student’s *t* test. **P < 0.01, ***P < 0.001. (**F**) Confocal images of *myl7*:RFP^+^ (labels cardiomyocytes red) and BRE:dsEGFP^+^ (green) embryos confirm odanacatib treatment improves BMP signaling in MLII embryos. Data quantified in Supplemental Figure 2. *n* = 10–15 embryos imaged from 2 experiments. Scale bar: 30 μm.

It was unclear from the BMV109 data whether MLII heart defects stem from the increased activity of Ctsk and Ctsl, or — conversely — whether reduced activity of Ctss is a phenotypic driver. Furthermore, these data do not clarify whether unregulated activation of Ctsk and Ctsl adversely affects Ctss’s activity. To begin addressing these questions, WT and MLII embryos were treated at 52 hpf with the highly specific Ctsk inhibitor odanacatib (50). The time point 52 hpf was chosen, since this time point precedes the final steps of VPC condensation and cardiac jelly “invasion,” both occurring closer to 60 hpf. Remarkably, confocal analyses of 4 dpf *tie2*:EGFP^+^ embryos stained immunohistochemically for myosin show that inhibiting Ctsk restores WT-like morphology to MLII hearts and valves in 58% of the embryos treated (Figure 5, A and B). This was true in both morphant and mutant embryos. Proper valve behavior and blood flow were also substantially improved, with 74% of the odanacatib-treated MLII valves opening and closing (“clapping”) with each contraction. A similar degree of rescue was noted using morpholinos to inhibit *ctsk*’s expression (Figure 5B). Odanacatib-mediated amelioration of the MLII phenotypes also corresponded with increased VPC differentiation, as assessed by in situ hybridization for *notch1b* expression. Unlike DMSO-treated embryos, 53% of the MLII embryos treated with odanacatib exhibited a *notch1b* pattern that was restricted to the valves (Figure 5C). Phenotypic rescue was associated with increased BMP signaling (Figure 5, D and E). Immunohistochemical analyses of pSmad1/5/8 demonstrated that, unlike MLII hearts, where very little pSmad1/5/8 activation was evident, odanacatib treatment increased the number of *gnptab*-deficient cells with nuclear localized pSmad1/5/8 by 45%. Increased staining was found throughout the atrial and ventricular chambers, with improvement particularly prominent in the right half of the ventricle. The intensity of pSmad1/5/8 staining in odanacatib treated hearts was even occasionally higher than that noted in WT hearts, suggesting a possible relationship between Ctsk activity and the level of BMP signaling. These findings were confirmed using the BRE:dsEGFP;*cmlc*:RFP–double transgenic animals (Figure 5F and Supplemental Figure 2).

**Figure 6:**
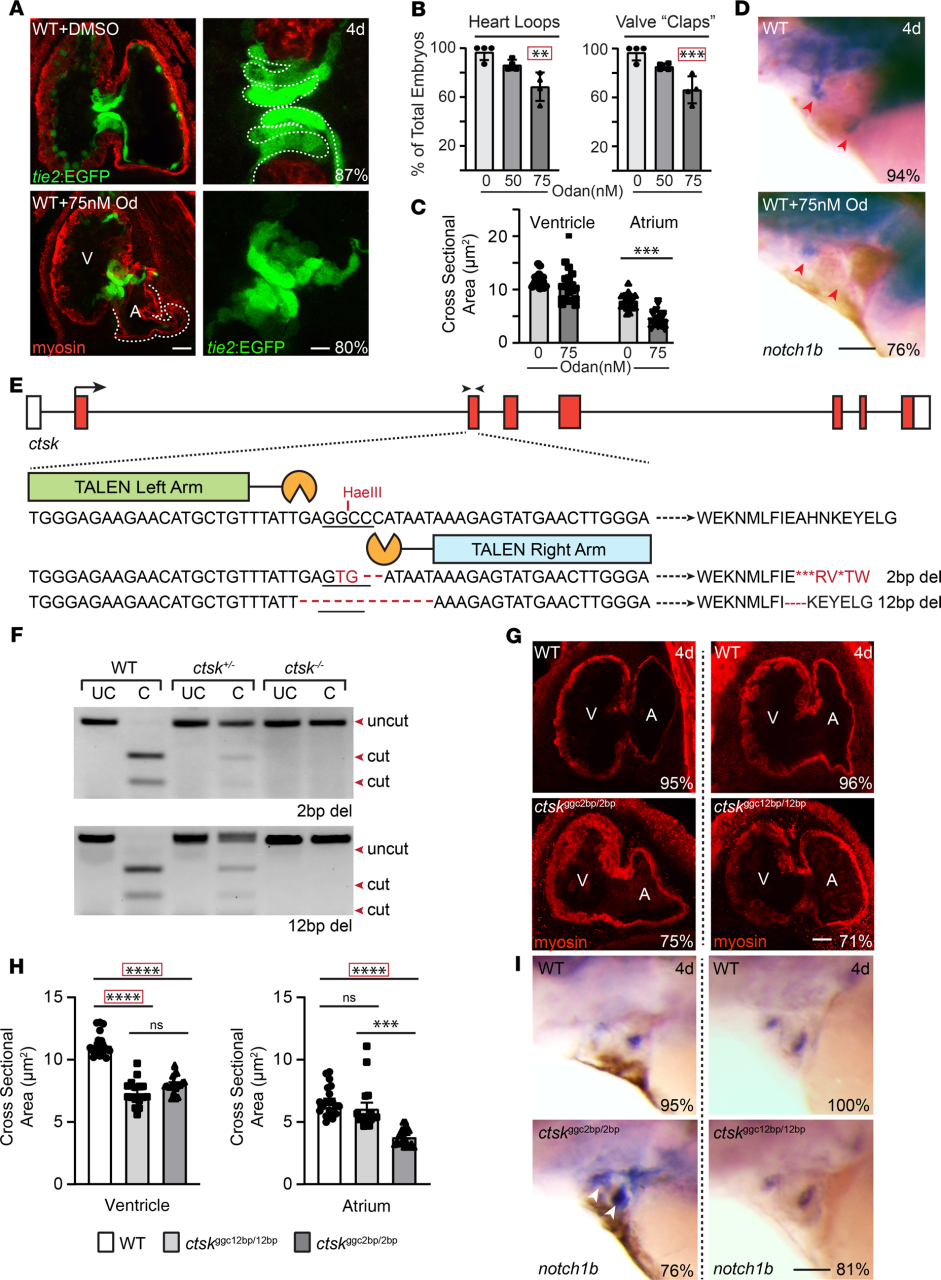
Ctsk deficiency alters valve and myocardial development. (**A**) Confocal images of *tie2*:EGFP embryos treated with odanacatib show alterations in both myocardia (red) and valves (green). In particular, the atria of Ctsk-inhibited hearts are smaller, and the valve cells are less organized. The white line denotes the defined layers present in the WT valve. Percent values represent the number of animals exhibiting these phenotypes. *n* = 20 embryos from 3 independent experiments. Scale bar: 30 μm (left panels) and 10 μm (right panels). (**B**) Quantification of the number of control and odanacatib-treated embryos whose hearts “loop” and valves open and close (“clap”) at regular intervals. Each dot represents an experiment with 25 embryos, with 100 total embryos scored. (**C**) The cross-sectional area of the ventricles and atria is shown from *n* = 15–20 embryos. Data are presented as mean ± SEM; **P < 0.01,***P < 0.001 by 2-tailed Student’s *t* test or Dunnett’s corrected test (denoted by red box). V, ventricle; A, atrium. (**D**) In situ analyses of *notch1b* expression show reduced expression in AV valves of odanacatib-treated embryos (red arrow heads). Percent values represent the number of animals exhibiting these phenotypes. *n* = 30 embryos from 3 independent experiments. Scale bar: 50 μm. (**E**) Schematic depicts TALEN targeted sequences in *ctsk*, resulting INDELS, and genotyping strategy. Black arrowheads denote position of genotyping primers. (**F**) Restriction enzyme–based genotyping of PCR amplified samples that were left “uncut” (UC) or “cut” with the HaeIII restriction enzyme. (**G**) Confocal images of WT and *ctsk* mutants stained immunohistochemically for myosin (using MF20, red) show reduced size of atria in embryos homozygote for the 2 bp–deleted allele. Percent values represent the number of animals exhibiting these phenotypes. *n* = 30 embryos imaged from 3 independent experiments. Scale bar: 30 μm. (**H**) quantification of cross-sectional area of ventricles and atria in WT and *ctsk*-compromised embryos. Data are presented as mean ± SEM. ***P < 0.001, ****P < 0.0001 using 2-tailed Student’s *t* test or Dunnett’s corrected test (denoted by red box). (**I**) In situ analyses of *notch1b* expression show its boundary is expanded in AV and OFT valves (white arrowheads) of *ctsk*^2bp/2bp^ embryos (red arrow heads). Percent values represent the number of animals exhibiting these phenotypes. *n* = 25–30 embryos from 3 independent experiments. Scale bar: 50 μm.

In addition to implicating Ctsk as a central driver of MLII cardiac pathology, these data highlight a normal role for the enzyme during early heart development. This is supported by the fact that heart looping and AV valve function were impaired in > 50% of WT animals treated with either odanacatib or a *ctsk* morpholino (Figure 6, A and B). Confocal images of *tie2*:EGFP^+^ embryos treated with 75 nM odanacatib show smaller irregular atria (Figure 6C). Like noted in *gnptab*-deficient MLII hearts, the valvular tissue in Ctsk-inhibited embryos was also less organized, with discrete cellular layers difficult to identify compared with control-treated embryos (Figure 6A). Although *notch1b* expression was restricted to the valvular regions, 76% of the embryos treated with odanacatib had reduced *notch1b* staining in AV regions (Figure 6D). Therefore, to further explore Ctsk’s impact during cardiovascular development, we used TALEN targeting exon 3 to create a *ctsk*-null line. TALEN-injected adults carrying 2 bp and 12 bp deletions were isolated and outcrossed over 5 generations with age-matched TLAB animals. Founders carrying mutations in *ctsk* were identified using HaeIII enzyme digestion of genomic PCR products generated from F1 outcross progeny (Figure 6, E and F). DNA fragments not cleaved by HaeIII were cloned and sequenced, and stable lines were generated. Enzyme digestion patterns were used to assign adult and embryonic genotypes in all subsequent generations. The progeny of *ctsk*^+/–^ matings were genotyped before analyses (Figure1D). As seen in odanacatib-treated embryos, the ventricles and atria in *ctsk*^–/–^ embryos carrying the 2 bp frame-shifting mutation (*ctsk*^ggc2bp/2bp^) were typically smaller than those of WT clutch mates (Figure 6, G and H). Interestingly, the ventricles (but not atria) from embryos homozygous for the 12 bp deletion, which maintains the reading frame but deletes 4 amino acids, were also small. Furthermore, the boundary of *notch1b* expression was expanded in 81% of the OFT and AV valves from *ctsk*^ggc2bp/2bp^-null embryos (Figure 6I). This is reminiscent of the loosely organized VPCs observed in embryos treated with odanacatib. Taken together, our data suggest Ctsk mislocalization compromises heart development in *gnptab-*deficient MLII embryos and further indicate that Ctsk activity may play a normal role in myocardial and valvular formation. Numerous other studies have also implicated Ctsk in heart and valve formation, but its role has been unclear partly because *Ctsk*-KO mice do not exhibit obvious defects in valvular form or function.

### Forced hypersecretion of Ctsk is sufficient to drive myocardial phenotypes.

**Figure 7:**
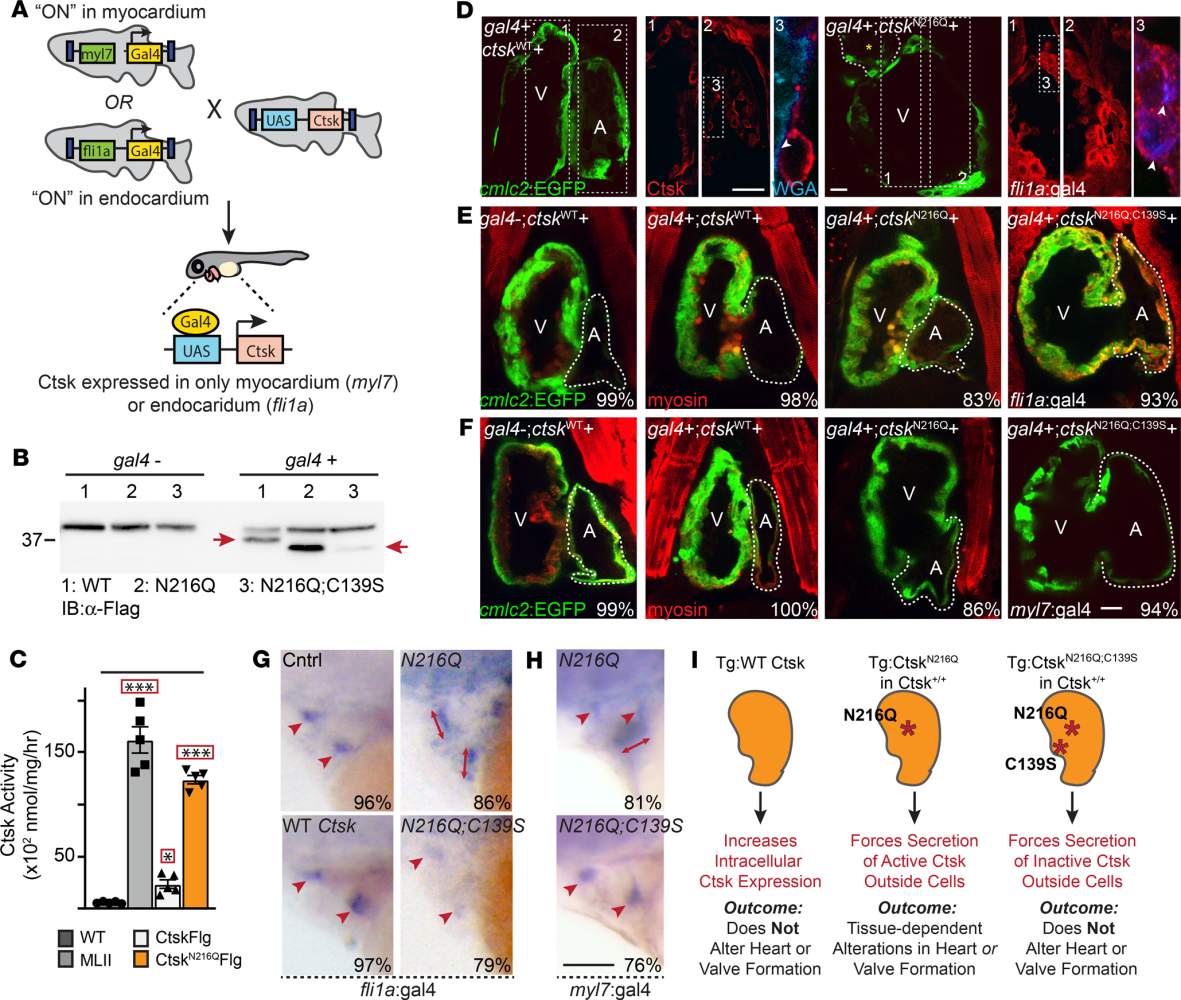
Forcing Ctsk hypersecretion disrupts heart and valve development. (**A**) Schematic illustrates Gal4-UAS bipartite expression system, which restricts expression of Flag-tagged Ctsk to either myocardial (driven by *myl7*:GAL4) or endocardial (driven by *fli1a*:GAL4) cells. (**B**) Immunoblotting with anti-Flag antibodies for 1:WT, 2:N216Q, 3: C139S;N216Q Ctsk transgenes in gal4^–^or gal4^+^ embryos show each is expressed and reveals increased protein stability and processing of hypersecreted (N216Q) Ctsk variant (red arrows denote cathepsin K) (**C**) Analyses of Ctsk activity in pools of 15 embryos show that, like MLII embryos, expression of the N216Q-secreted variant is associated with more enzyme activity than induction of the WT Ctsk transgene. Data are presented as mean ± SEM. **P < 0.01. ***P < 0.001 using Dunnett’s test (denoted by red box). *n* = 4 experiments. (**D**) Confocal analyses of sections of embryos expressing WT or N216Q-secreted forms of Ctsk stained for the transgene (red) and extracellular matrix (WGA, blue) show WT Ctsk is retained within cells (as it is distinct from WGA stain), while the N216Q variant is secreted. This is illustrated by overlap with extracellular WGA (white arrowheads). Panels 1, 2, and 3 represent higher-power views of regions of interest. (**E**) Confocal analyses of 4 dpf hearts in progeny of *fli1a*:gal4 and UAS:*ctsk* parents expressing either WT, N216Q-, or C139S;N216Q-secreted forms of Ctsk. The genotype with regard to gal4 (+ or –) and *ctsk* is denoted on each panel. Embryos are *cmlc2*:EGFP^+^ (green) and stained immunohistochemically for myosin (red). Images show forcing secretion of Ctsk (N216Q) from endocardial cells does not alter heart morphology, although atria are often slightly smaller. (**F**) Confocal analyses of 4 dpf hearts in progeny of *myl7*:gal4 and UAS:*ctsk* parents. The genotype of gal4 (+ or –) and *ctsk* (WT, N216Q, or C139S;N216Q) is denoted. Images show that forcing secretion of Ctsk (N216Q) from myocardial cells alters heart morphology. *n* ≥ 30 embryos from 4 experiments. Scale bar: 20 μm. (**G** and **H**) In situ analyses of *notch1b* in animals expressing WT or a cathepsin K variants (N216Q or C139S;N216Q) secreted from endocardia (**G**, *fli1a*:gal4) or myocardia (**H**, *myl7*:gal4) show secreting *active* Ctsk from either tissue impairs valve cell differentiation (arrow heads versus expanded arrows). Control animals are gal4^–^. Percent values represent the number of animals exhibiting phenotypes. *n* = 30–35 embryos from 3 experiments. Scale bar: 50 μm. (**I**) Schematic summarizing outcomes of Gal4-UAS experiments. V, ventricle; A,atrium.

To independently assess whether inappropriate secretion of Ctsk alone impairs myocardial development, we generated transgenic zebrafish lines that conditionally express flag-tagged forms of the protease under 2 different cardiac promoters (i.e., *cmlc* and *fli*) (Figure 7 and Supplemental Figure 3). Because increasing Cts activity may cause lethal cardiovascular defects, expression was controlled using the bipartite Gal4–upstream activation sequence (Gal4-UAS) inducible system, in which 1 fish line carries a UAS-flanked transgene for the tagged Cts and a second fish line carries a transgene with a tissue-specific promoter that controls expression of the Gal4 activator (51). Using this method, cathepsin expression is only activated in progeny when 2 lines are crossed (Figure 7A). We generated UAS lines expressing either WT *ctsk* or a *ctsk* variant lacking one of the enzyme’s 2 N-glycans. Loss of this N-glycan and the M6P residues it would ultimately bear impairs intracellular sorting and forces Ctsk’s hypersecretion. We also generated an enzymatically inactive version of this secreted variant, C139S;N216Q. We have demonstrated by transfection in cell culture and RNA injection in zebrafish that these mutant forms are expressed and that WT Ctsk-Flag and the N216Q variant are enzymatically active (Figure 7, B and C). As previously noted in MLII embryos, mutation of the C-terminal N-glycan (N216Q) on zebrafish Ctsk yielded a 2-fold increase in its enzymatic activity. In parallel, assays with the BMV109 ABP the C139S;N216Q “catalytically dead” double mutant exhibit no activity relative to background (Supplemental Figure 4) (20, 32). Increased activity of the N216Q variant corresponded with enhanced processing of inactive ProCtsk (Figure 7B). Although less stable, the inactive C139S;N216Q variant showed similarly increased processing. These data are consistent with previous findings from MLII embryos, which suggested that, when localized outside the cell, increased processing of ProCtsk is associated with its increased activity (21). Immunohistochemical analyses of the flag epitope in hearts expressing either WT Ctsk-Flag or the N216Q variant show that Ctsk^N216Q^ (but not WT) is indeed secreted from myocardial (*myl7*:gal4) and endocardial cells (*fli1a*:gal4) (Figure 7D). This is demonstrated using wheat germ agglutinin (WGA) as a marker for the extracellular boundary. Unlike controls where WT Ctsk staining (red) is clearly distinct from WGA (blue), stains of the N216Q variant extend beyond the cell overlapping with extracellular WGA (white arrowheads) (Figure 7D and Supplemental Figure 4).

Confocal analyses of 4 dpf hearts stained immunohistochemically for myosin show that inducing expression of WT Ctsk in either myocardial or endocardial cells does not notably alter heart morphology or function (Figure 7, E and F). In contrast, depending on the tissue from which it was secreted, expression of the secreted N216Q variant caused several phenotypes. Defects in heart morphology, including incomplete looping and dilated ventricles, were primarily noted in *myl7*:GAL4;Ctsk^N216Q^–positive embryos where Ctsk is secreted from myocardial cells. Deflated small atria were seen in both *fli1a*:GAL4;Ctsk^N216Q^–positive and *myl7*:GAL4;Ctsk^N216Q^–positive embryos. In situ analysis of *notch1b* expression confirmed that, regardless of the originating tissue, inappropriate secretion of Ctsk also disrupts valve formation (Figure 7, G and H). As previously demonstrated, *notch1b* transcripts were restricted to the OFT and AV valves in 85% of control embryos and 91% of those expressing the Ctsk^WT^ transgene. However, 47% of the embryos expressing the Ctsk^N216Q^ from endocardial (driven by *fli1a:GAL4*) and 52% from myocardial cells (driven by *myl7:GAL4*) show expanded *notch1b* expression in the AV and outflow tract valves. These data show that inappropriate secretion of Ctsk is sufficient to disrupt VPC maturation. Expression of the catalytically inactive variant (Ctsk^C139S;N216Q^) did not expand *notch1b e*xpression, but its levels were reduced in *fli1a*:GAL4;Ctsk^C139S;N216Q^–positive embryos (Figure 7G). These data suggest that simply increasing intracellular expression of Ctsk does not alter heart development, but its mislocalization outside cells can impact myocardial, endocardial, and valvular tissue formation (Figure 7I).

## Discussion

The role for cathepsin proteases in early heart and valve development is still not clearly defined, but analyzing cardiac systems in models of inherited disease can offer valuable insight into these processes (24). Here, we show that mutations in the *gnptab* gene, associated with the lysosomal disease MLII, alters the activity and localization of cysteine cathepsins and disrupts heart and AV valve development in zebrafish embryos. Using a TALEN-mediated KO of *gnptab*, we demonstrate that secretion of cathepsin K not only increases its activity within the developing heart, but also drives local alterations in TGF-β–related signaling. This is supported by the findings that treatment with the cathepsin K–specific inhibitor odanacatib restores normal signaling profiles, improving heart and valve morphology and overall cardiovascular function. WT animals treated with odanacatib also exhibited defects in heart and valve morphology, providing evidence that cathepsin K is important for normal cardiovascular development. The implications of these findings in the context of the cellular and molecular pathways that govern cardiovascular development are discussed, along with the potential of cathepsin inhibition as a therapy for MLII.

Defects in cardiovascular development and homeostasis have been noted in several animal models of LSDs, especially the mucopolysaccharidoses (52, 53). Analyses of affected tissues have shown abnormal expression of both the matrix metalloproteinases and cathepsin proteases, but establishing whether these enzymes contribute to heart pathology has been difficult. Alterations in cathepsin B have been observed in the diseased valves of MPSVII dogs, as well as the hearts of MPSI mice (53). Baldo and coworkers further demonstrated that inhibiting cathepsin B in MPSI mice improves but does not completely normalize cardiac function (52). In both studies, the basis for increased cathepsin expression was not clear but was suggested to possibly arise from immune activation in these tissues (52, 54). The present work defines a pathogenic role for cathepsin K, uncovering insight into the basis for its increased activity by demonstrating that enzyme secretion itself enhances processing and activation. This pathogenic scenario appears distinct from previously described MPS disorders, as it is mediated at the level of localization and activation — not abnormal expression. This is supported by prior work that found no difference in *ctsk* transcript abundance in MLII zebrafish embryos, supporting a posttranscriptional mode of enzyme dysregulation (32).

Unlike the prior studies that implicate altered extracellular matrix (ECM) remodeling as the source of MPS cardiac disease (52–54), in MLII, the downstream effects of spurious cathepsin activity are largely driven by altered growth factor signaling. In fact, because ECM production is less abundant in valvular tissue from 48 to 72 hpf, cathepsin K’s normal role during at these early stages may extend beyond its ECM-degrading activities (55). Our data show that cathepsin K inhibition restores BMP signaling and normal heart and valve development in *gnptab-*deficient embryos, suggesting that inappropriate cathepsin K secretion adversely affects growth factor stability or activation in the heart. Cathepsin K–mediated alterations in TGF-β and BMP signaling were previously demonstrated in developing cartilage and bone, where cathepsin K secretion also enhanced TGF-β activity, disrupting cellular differentiation (20, 21). Here, we link cathepsin K secretion with aberrant cardiovascular development, suggesting that disrupted TGF-β–related signals impair heart morphogenesis and valve cell differentiation. This leads to a loose valve structure that is unable to open and close as the heart contracts, causing blood to regurgitate and pool at the heart’s base. Multiple groups have considered cathepsin K’s role in cardiovascular development, showing that it is not only expressed in developing endocardia, but also that cathepsin K activates Notch1 during VPC differentiation (56–59). These are compelling findings, given Notch1’s importance for valvular development and our findings that *notch1b* expression is altered when cathepsin K expression, activity, or localization is disrupted. Our analyses of cathepsin K’s function in the context of normal development, as well as MLII pathogenesis, provide evidence that disrupting cathepsin K localization or activity alters heart and valve formation.

The pathogenic contribution of cathepsin K is reinforced by studies using transgenic embryos that express a secreted variant lacking one of the enzyme’s 2 N-glycans. Like noted in MLII embryos, loss of this glycan causes cathepsin K to be secreted, which increases its activity and disrupts myocardial morphology and valvular development. These effects occur despite normal intracellular levels of protease. Studies with a catalytically inactive form of cathepsin K confirmed that its extracellular pathogenicity depends on enzymatic activity. Like noted in MLII embryos, increased cathepsin K activity results from enhanced processing of the enzyme’s “pro” form. Intriguingly, secretion of inactive cathepsin K was also associated with increased protein processing, but the inactive enzyme was highly unstable. Earlier studies in MLII embryos indicate that extracellular processing, increased activity, and enhanced stability of the secreted enzyme depend on its interaction with chondroitin sulfate glycosamino glycans, which are enriched in the valvular matrix (20, 21). The high chondroitin sulfate content present in the heart valve may make it particularly sensitive to mislocalized cathepsin K.

Importantly, severity of the cardiac phenotypes associated with mislocalized cathepsin K depended on the tissue from which it was secreted. When secreted from endocardial tissue, cathepsin K predominantly affected valvular development, while secretion from myocardium impaired both heart and valve morphology. Since forced secretion of Ctsk from endocardial cells impaired valvular development in the absence of obvious myocardial defects, this phenotype is not likely secondary to altered myocardial formation. These data also indicate that, once outside the cell, the protease does not diffuse far and primarily impacts substrates close to the tissue of origin. Since BMPs are secreted from myocardial cells and TGF-β from endocardial cells, our data imply that defective valvular development associated with MLII may primarily stem from disrupted TGF-β signaling, while altered heart morphology noted in zebrafish embryos more likely stems from disrupted BMP signaling. Because MLII patients predominantly exhibit mitral valve deficiency and aortic regurgitation, inhibiting TGF-β signaling may be a viable intervention for valvular disease in LSDs like MLII. This is supported by the findings that treatment with an inhibitor of TGF-β signaling improved valvular morphology in MLII zebrafish. Collectively, these data provide fundamental information regarding the pathogenic consequences of cathepsin K secretion in the developing heart and highlight the potential of small molecules (namely cathepsin inhibitors and growth factor modulators) as viable therapeutic strategies. Successful treatment of aortic disease using losartan to reduce TGF-β signaling in the context of Marfan’s syndrome provides support for this possibility (60). It is currently unclear why the increased activity of secreted cathepsin K and reduced cathepsin K activity or expression alters early heart development in a similar ways. The fact that our studies with the hypersecreted variant were performed in an otherwise normal background (normal intracellular Ctsk) suggests the resulting phenotypes are not due to loss of intracellular enzyme. Rather, these findings argue that Ctsk normally influences TGF-β signaling and any disruption to either its expression or localization impacts this process. Systematic analysis of pharmacological approaches to modulate this within available animal models of MLII will be an important focus for future studies.

## Methods

### Zebrafish strains and husbandry.

Animals were maintained according to standard protocols. The TL, AB, BRE:EGFP (61), and *Tg(fli1a:EGFP)^y1^* (62) zebrafish strains were obtained from the Zebrafish International Resource Center (ZIRC). The *cmlc2:*RFP and *cmlc2:*EGFP strains were provided by Caroline Burns (Harvard Medical School; Boston, Massachusetts, USA) (63), and the SBE:EGFP line was provided by Enrico Moro (Padova, Italy) (64). Embryonic staging was performed according to established criteria (65). In some cases 0.003% 1-phenyl 2-thiourea (PTU) was added to embryo medium to block pigmentation.

### Morpholino knockdown and generation of gnptab TALEN mutants.

Morpholino knockdown of GNPT was performed and assessed as previously described (33). Capped and polyadenylated mRNA of TALEs targeting *gnptab* was produced using Message Machine (Thermo Fisher Scientific). *gnptab* TALEN-KO mutant zebrafish were generated by coinjecting 25 pg of mRNA for each of the right and left TALE arms into WT (TLAB hybrid) embryos at the 1-cell stage. Similarly, *ctsk-*null animals were generated following injection of 50 pg of mRNA for each of the right and left TALE arms. Embryos were grown to adulthood, and potential F0 founders were screened as described in the results section; stable lines were generated as described in the results section.

### Confocal imaging of live and fixed embryos.

For analyses of BMP signaling, BRE:dsEGFP and BRE:EGFP transgenic embryos were manipulated (either via morpolino injection or pharmacological treatment) as indicated in Results. Embryos were ultimately anesthetized with 0.4 mM Tris-buffered MS-222 (MilliporeSigma) and mounted on coverslips in hanging drops of 0.8% low-melt agarose as previously described (66). Fixed and immunohistochemically stained embryos were similarly mounted for microscopic imaging. Coverslips were placed across the central well of standard 60 mm organ culture dishes containing Danieus growth media. Animals were imaged with a 40× water immersion objective (NA 1.15) on an Olympus FV3000 LSM confocal microscope. All levels were chosen according to optimal imaging parameters, such that signals were not saturated and settings were kept identical between sample types. Maximum intensity projections were generated with ImageJ software (Open Source, NIH), and images were subsequently processed with Adobe Photoshop (CS6 extended, version 13.0). For images requiring 3D rotations, initial processing was performed using the Slidebook package 7.0 (3i).

### In situ hybridization.

Analyses were performed according to Thisse and Thisse protocol (67). The DNA construct used to generate the *notch1b* probe was provided by Barry Paw (Harvard Medical School). The construct was linearized with BamHI and T3 RNA polymerase used to generate the probe. An *spp1*probe template was generated by PCR from 3 dpf embryonic cDNA and T7 RNA polymerase used to create the probe. The primers were: forward, 5′ - AGCGACTACAAAAAATCCATCGTCT - 3′; reverse, 5′ - TG TAATACGACTCACTATAGGG CTGAACAAGTTTGGCAGCAGTTCGA - 3′.

### IHC whole mounts and sections.

For analyses of Ctsk localization, 20 μM sections from *fli1a*:EGFP^+^ hearts were stained immunohistochemically with Alexa-647 conjugated WGA (1/200), an anti-Flag antibody (MilliopreSigma, F7425, 1/1000), anti-Ctsk (Abcam, ab19027, 1:100), anti-Ctss (R&D, AF1183, 1:100), ant-GFP (Invitrogen, A-11120 or A-11122, 1:200), and myosin (MF20, Developmental Studies Hybridoma Bank, 1/100). Appropriate Alexa Fluor–conjugated secondary antibodies were used for visualization. For whole mount analyses of heart morphology, embryos were stained for myosin (MF20, Developmental Studies Hybridoma Bank, 1/100). Antibodies for activated Smads were pSmad2 (Cell Signaling Technologies, 8828, 1:200) and pSmad1/5/8 (Cell Signaling Technologies, 9511, 1:200). In some cases, hearts were also visualized using a *cmlc*:EGFP transgene associated with the UAS transgenes. Samples were imaged on an FV3000 laser scanning confocal microscope with a 40× (N.A.1.15) water immersion lens. All measurements and quantitative analyses were performed using the measurement tools in the CellSense software on the Fluoview System. Image projections and 3D reconstruction/rotations were generated with either ImageJ (NIH) or Slidebook 5.0 (3i) software. Final images were processed in Adobe Photoshop Extended.

### BMV109 delivery and embryo labeling.

The BMV109 ABP was injected into embryos as previously described (21). Briefly, 1 nL of a 10 μM solution of probe was introduced pericardially via microinjection. The probe was circulated overnight at the normal growth temperature (28.8°C), and embryos were imaged on an FV3000 confocal microscope 12 hours after injection.

### Western blot analyses.

Embryos were manually deyolked and harvested at the time points indicated. Lysates were generated as previously described (21). Western analyses for Ctsk transgenes were performed using an anti-Flag antibody (MilliporeSigma, F7425, 1/800) and HRP-conjugated secondary antibody. Blots were analyzed using the Bio-Rad MP Chemidoc system. Total protein load as assayed by Ponceau S staining was used to ensure equivalence of loading between lanes.

### Pharmacological inhibition.

TGF-β signaling was inhibited in live embryos by introducing the indicated concentration of SB505124 (Selleckchem, solubilized in DMSO) directly into their growth media at 52 hpf. For inhibition of Ctsk, embryos were also treated with the indicated concentration of Odanacatib (Selleckchem, solubilized in DMSO) at 52 hpf. Embryos were treated for 1–2 days. In all cases, WT control embryos were treated with an equivalent amount of DMSO (0.1%). For all treatment experiments, embryos were staged and synchronized by age within the first hours after fertilization and again before 24 hpf.

### FACS and qPCR.

Embryos were dissociated into single cells, and the EGFP^+^ population was isolated by FACS as previously described (32). Cells were sorted for purity to ensure no contamination of EGFP^–^ cells. To ensure high-quality RNA, cells were collected directly into TRIZOL reagent. RNA was isolated as previously described (32). qPCR was performed in accordance with the Minimum Information for Publication of Quantitative Real-Time PCR Experiments (MIQE) criteria and as previously described (32). The identity of the myocardial and endocardial cell population was confirmed using *tie2* and *cmlc* gene expression. A complete list of primers used can be found in Supplemental Figure 1. Amplified products were subsequently run on agarose gels.

### Statistics.

In cases where numerical or quantitative data were generated, SDs and 2-tailed Student’s *t* tests were used to assess statistical significance. In cases where multiple treatments were compared with a single control, the Dunnett’s test with correction was used. This is indicated in the figure legends. *P < 0.05, **P < 0.01, ***P < 0.001. Data were processed on GraphPad Prism (Version 7.0a). In cases where staining patterns were assessed visually, representative embryos are shown and the number of animals from multiple experimental samples that resembled those pictured was calculated. Embryo sex is not established until later in development and, as such. is not a relevant consideration for these studies.

### Study approval.

The Greenwood Genetic Center is a Public Health Service–certified institution. Handling and euthanasia of fish for all experiments complied with the policies of the Greenwood Genetic Center, as approved by the GGC’s IACUC (permit no. A2019-01-003-Y1-A1).

## Author contributions

PL, TM, and CJC performed experiments and interpreted data. TCL provided the DNA constructs containing TALEN sequences used to generate *gnptab*-KO lines. RAS assisted with data interpretation and manuscript preparation. HFS designed and performed experiments, interpreted data, and wrote the manuscript.

## Supplementary Material

Supplemental data

## Version 1 10/15/2020

Electronic publication
