# Supplementary material for: Inappropriate cathepsin K secretion promotes its enzymatic activation driving heart and valve malformation
Source: JCI Insight. 2020 Oct 15;5(20):e133019. doi: 10.1172/jci.insight.133019 (PMC7605527; doi:10.1172/jci.insight.133019)
Supplement: Supplemental data [file jciinsight-5-133019-s021.pdf]

A

Table of F0 founder animals outcomes

Line	Female	Male	Concentration Tale mRNA (pg)	Genetic Mutation	% F1 outcross progeny with Mutation
1	F		0.5 pg	5bp del	11/46
3*	F		0.5 pg	2bp del	14/14
5	F		0.5 pg	1bp del	1/20
6	F		0.5 pg	25bp ins; 8bp del	18/20
8	F		0.5 pg	5bp ins; 6bp del	15/20
10	F		0.3 pg	8bp del	4/20
14	F		0.3 pg	7bp del	2/20
19	F		0.3 pg	4bp ins; 3bp ins	9/20
1		M	0.5 pg	5bp del; 8bp del	2/11
2*		M	0.5 pg	5bp del	17/20
3*		M	0.5 pg	2bp del; 7bp del	13/13
5		M	0.5 pg	4bp del; 5bp del	7/14
6		M	0.5 pg	5bp del; 8bp del; 10bp ins	6/15
9		M	0.5 pg	1bp del	2/20
10		M	0.5 pg	1bp ins	4/20
13		M	0.3 pg	6bp del	10/21
14		M	0.3 pg	5bp del;7bp del	5/20

B

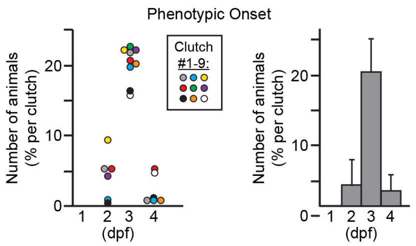

C

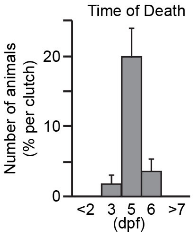

D

Table of primers used for RT-PCR analyses and genotyping

Primer Name	Primer Sequence	Gene Location	Experimental Use
gnptab TALE For:	TATGCGGCATATCAGACTTGA	Intron 7 to Intron 8	RFLP genotyping
Rev:	CTTCAATTCACAAACATCCCAT		
gnptab TALE For:	AATGGAGTCGAGATCAGTACCA	Ex2 to Intron2	HRM genotyping
Rev:	TCAACCACTCACCTCGTCTG		
gnptab For:	TATGCGGCATATCAGACTTGA	Ex7 to Ex8	RT-PCR and Sequencing
Rev:	CTTCAATTCACAAACATCCCAT		
ctsk TALE For:	ATGAATTCTAACAATTTGTCCGTGC	Ex3 to Ex3	RFLP genotyping
Rev:	AACTTGGGTGAGTTTGTAACAATAC		
ctsk For:	GGATCAGCAGTGTGCCTACA	Ex4-5 to Ex5	Traditional PCR genotyping
Rev:	TGGACTGCATGGCATCTATG		
cmlc2 For:	GGAGAGAAGCTCAATGGCACA	Ex4-5 to Ex6	Quantitative and Non-Quantitative RT-PCR
Rev:	GGGTCATTAGCAGCCTCTTG		
ctsla For:	GGAGGGTCAGATGTTCAGGA	Ex5 to Ex5	Quantitative and Non-Quantitative RT-PCR
Rev:	CAGTCCACCAGGTTCTGCTC		
ctss1 For:	ACTGCACTGGCTTCAGGATT	Ex6 to Ex6	Quantitative and Non-Quantitative RT-PCR
Rev:	GCCCGATGTTAGCGACAG		
tie2 For:	AGCACACTCTCCTCACAGCA	Ex17-Ex18	Quantitative and Non-Quantitative RT-PCR
Rev:	TTCGCCACAAAGTTCTCTCC		
rpl4 For:	GTGCCCGACCGTTAATCTC	Ex2 to Ex2	Quantitative and Non-Quantitative RT-PCR
Rev:	ACACTGCTGGCATAACCACAT		

Supplemental Figure 1. A) Table illustrating the outcomes of F0 founder analyses. 20 males and 20 females were screened following injection of indicated TALEN concentrations. Shown are animals with interesting insertions and deletions following sequence analyses. The genetic mutations detected and the number of offspring from outcross matings with TLAB carrying the mutation listed. F1 outcrossed lines were generated from female 3 and males 2 and 3. B) Graph illustrates timing of phenotypic onset as scored from 9 distinct clutches. Circles represent individual clutches and indicate the number of embryos in the clutch (y axis) and time of phenotypic onset (x axis). This is also shown in the accompanying bar graph. C) Graph illustrates time of death for embryos in these phenotypically scored clutches. D) List of primers used in the study.

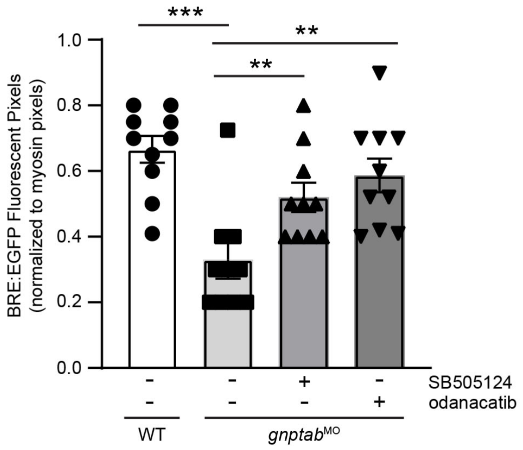

Supplemental Figure 2. BMP signaling is reduced in the hearts of *gnptab*-deficient MLII embryos. A) Graphs represent quantitation of BMP signaling, as assessed using BRE:EGFP transgenic zebrafish. EGFP fluorescence was calculated in the right half of the ventricle. EGFP fluorescence was normalized to fluorescent units for myosin staining within the same region.

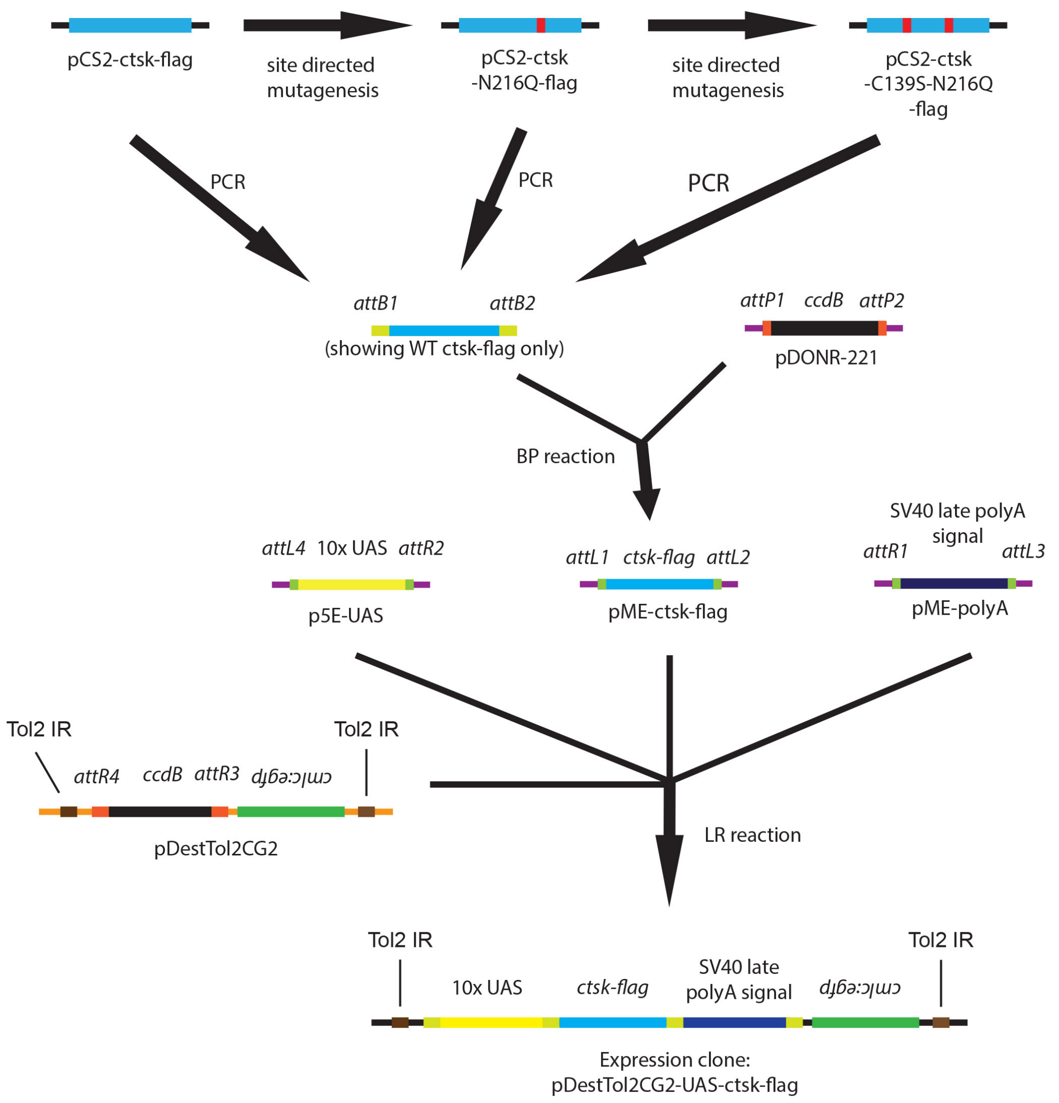

Supplemental Figure 3. Schematic illustrating generation of TOL2 constructs to create transgenic zebrafish lines expressing WT, N216Q, and N216Q;C139S forms of cathepsin K under the control of UAS.

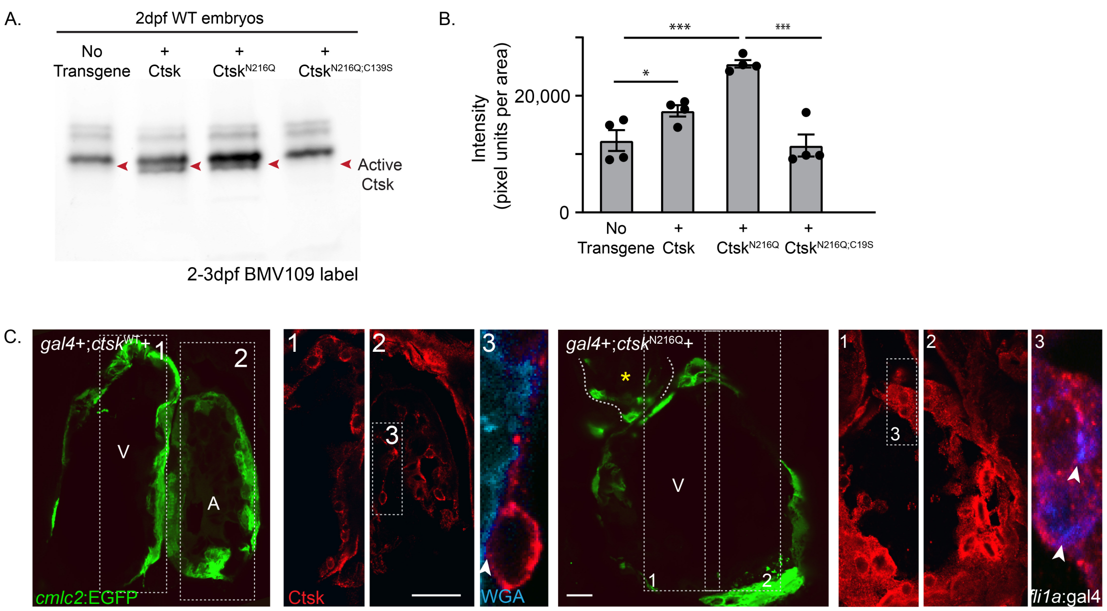

Supplemental Figure 4. A) BMV109 labeling of all transgenic conditions show Ctsk activity is increased in the CtskN216Q variant but completely lost in the $\text{Ctsk}^{C139S;N216Q}$, suggesting the variant is indeed catalytically dead. Representative gel from 4 independent experiments. B) Densitometric quantitation of Ctsk activity in 4 experiments. All measurements normalized to total protein load as assayed by Coomasie stain following gel imaging. C) Enlarged images from from Figure 7D showing the N216Q Ctsk variant is secreted from endothelial cells (see white arrows, panels 3). This is demonstrated by overlap with WGA (blue), which labels glycans at the cell surface and in the extracellular space. This is in contrast to WT Ctsk which is clearly distinct from extracellular WGA. Scale bars=20μm.
